# Massive hepatic necrosis-associated acute liver failure

**DOI:** 10.1136/egastro-2025-100217

**Published:** 2026-01-09

**Authors:** Tao Lin, Chenhao Tong, Roman Liebe, Honglei Weng

**Affiliations:** 1 Department of Medicine II, Section Molecular Hepatology, University Medical Center Mannheim, Medical Faculty Mannheim, Heidelberg University, Mannheim, Germany; 2 Hepatopancreatobiliary Surgery Department, The First Affiliated Hospital of Ningbo University, Ningbo, Zhejiang, China; 3 Klinik für Gastroenterologie, Hepatologie und Transplantationsmedizin, Universitätsklinikum Essen, Essen, Germany

**Keywords:** Liver Diseases, Acute Liver Failure, Massive Hepatic Necrosis, Liver Regeneration, Cellular Microenvironment

## Abstract

Massive hepatic necrosis (MHN), characterised by extensive loss of hepatocytes, represents the most severe pathological lesion in acute liver failure (ALF). MHN-associated ALF primarily occurs in patients with acute viral hepatitis A, B or E infections. Additionally, MHN-associated ALF can develop in patients with autoimmune hepatitis or in those taking idiosyncratic drugs. MHN-associated ALF is associated with significantly higher mortality than that caused by other aetiologies. In contrast to other forms of ALF, liver regeneration following MHN depends on liver progenitor cells (LPCs)—the smallest cholangiocytes localising in the canal of Hering and terminal biliary branches. These cells play key roles in determining the clinical outcome of patients with MHN-associated ALF. This paper reviews the pathophysiology of MHN-associated ALF and recent advances in LPC biology.

 Acute liver failure (ALF) is a life-threatening clinical syndrome characterised by rapid deterioration of liver function, coagulopathy and hepatic encephalopathy in the absence of pre-existing liver disease.^1^ The commonly accepted ALF definition was coined by O’Grady and colleagues in 1993.^2^ In their paper, they proposed the term ‘acute liver failure’ for cases within an interval of between 8 and 28 days from jaundice to encephalopathy; these cases also exhibit a high incidence of cerebral oedema and a much poorer prognosis without liver transplantation.^2^ To date, ALF is defined as a severe liver injury, leading to coagulation abnormalities (with an international normalized ratio (INR) ≥1.5) and any degree of mental alteration (encephalopathy) in a patient without pre-existing liver disease and with an illness of up to 4 weeks’ duration.^3 4^ Table 1 shows the evolution of the ALF concept and definition.

**Table 1:** Evolution of the concept and definition of ALF

Authors and time	Definition	Characteristics	Comments
Lucké B Lucké B, Mallory T^12 68^	Fulminant/fatal epidemic hepatitis	A rapidly fatal form of epidemic hepatitis with a clinical course of less than 10 days. Characterised by massive hepatic necrosis, minimal or absent jaundice, early onset of hepatic encephalopathy and profound liver cell destruction in the absence of pre-existing liver disease.	A historically important study for its indepth description of a formerly poorly characterised liver disease
Trey C, Davidson CS^13^	FHF	FHF was introduced to describe a potentially reversible condition, the consequence of severe liver injury, with an onset of encephalopathy within 8 weeks of the appearance of the first symptoms and in the absence of pre-existing liver disease.	The first definition of FHF/ALF
O'Grady JG *et al*^2^	ALF	The King’s College standard subdivided ALF into hyperacute ALF (encephalopathy within 7 days of the onset of jaundice), acute ALF (8–28 days) and subacute ALF (5–12 weeks) according to the interval from jaundice to encephalopathy.	The first official nomenclature of ALF
IASL^69^	Acute hepatic failure	Acute hepatic failure is a potentially reversible, often sudden, persistent and progressive liver dysfunction (in the absence of pre-existing liver disease) characterised by the occurrence of encephalopathy within 4 weeks from the onset of symptoms. Encephalopathy remains the most acceptable diagnostic feature of acute hepatic failure.	A definition proposed by the IASL
AASLD^70^	ALF	The most widely accepted definition of ALF includes evidence of coagulation abnormality, usually an INR ≥1.5 and any degree of mental alteration (encephalopathy) in a patient without pre-existing cirrhosis and with an illness of <26 weeks duration.	A definition proposed by AASLD
EASL^4^	ALF	Acute (fulminant) liver failure is defined as a rare syndrome in patients with no underlying chronic liver disease, characterised by acute abnormal liver tests (severe hepatocellular injury) plus coagulopathy of liver origin (eg, INR >1.5) and clinically apparent hepatic encephalopathy.	A definition proposed by EASL

AASLD, American Association for the Study of Liver Diseases; ALF, acute liver failure; EASL, European Association for the Study of the Liver; FHF, fulminant hepatic failure; IASL, International Association for the Study of the Liver; INR, international normalized ratio.

Over the last four decades, the epidemiology, clinical phenotype and disease course of ALF have been extensively investigated. To date, complex critical care protocols have been proposed and applied in clinical practice. For patients with severe ALF, emergency liver transplantation is still the only life-saving treatment option.^1 5 6^ A multitude of reviews have described the progress in the clinical management of ALF.^15^,^9^ ALF originates from severe liver injury induced by different aetiologies. Histologically, ALF results from two catastrophic events in the liver: (1) loss of the majority of the parenchyma, including massive hepatic necrosis (MHN) or submassive hepatic necrosis (SMHN) and (2) metabolic dysfunction in hepatocytes caused by mitochondrial toxicity.^6 10^ Both MHN and SMHN are morphological features of ALF (formerly termed fulminant liver failure).^11^ In liver pathology textbooks, MHN has been defined as extensive, diffuse panlobular (panacinar) and multilobular necrosis >60–70% of the entire liver on examination via explant, autopsy or clinical visualisation. In contrast, SMHN describes lesions with global necrosis of 30–70% of the entire liver.^11^ According to this definition, the difference between MHN and SMHN is reflected solely in the necrotic areas (figure 1). Notably, MHN can evolve into SMHN over time. In ALF cases with a clinical course exceeding 1–2 months, areas of necrosis become significantly reduced due to regeneration. Lefkowitch classified these cases as SMHN.^10^

**Figure 1:**
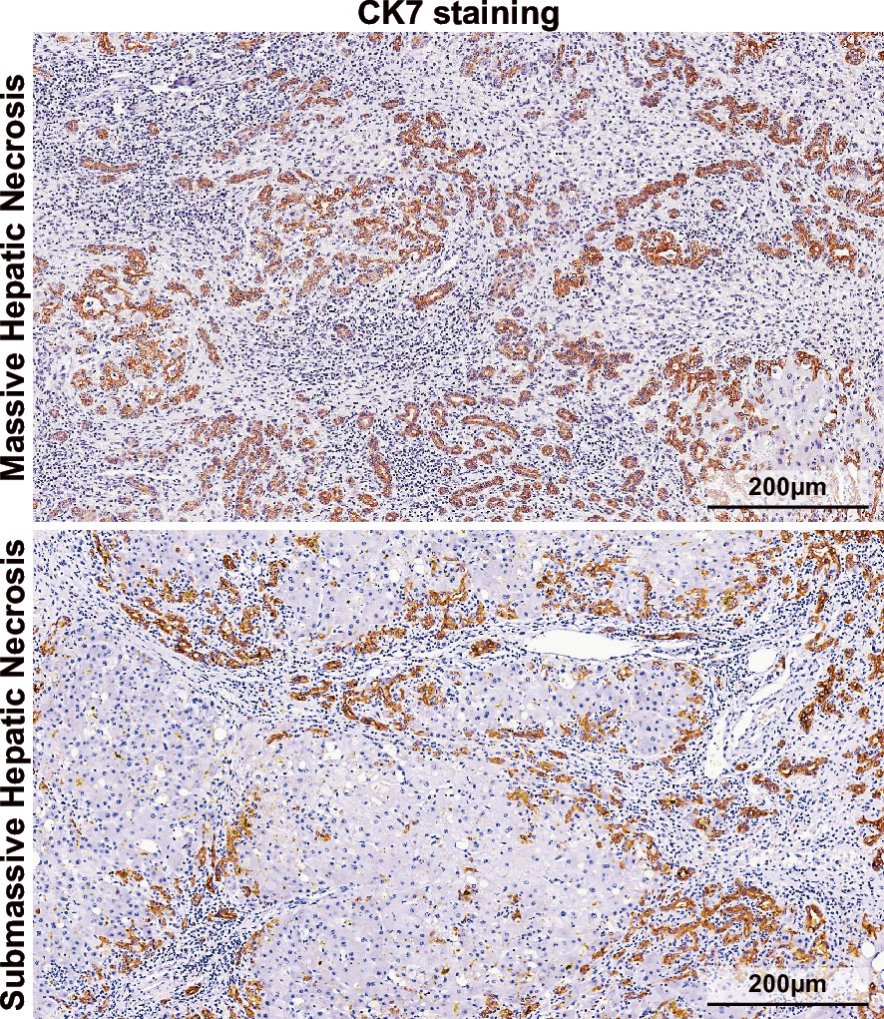
MHN and SMHN are observed in two patients with acute liver failure associated with HBV infection. Compared with SMHN, the liver damaged by MHN demonstrates more severe inflammatory cell infiltration, resulting in more extensive ductular reaction highlighted by immunohistochemical staining for CK7 (CK7 antibody, DAKO, M7018; stained by DAKO EnVision kit). CK, cytokeratin; HBV, hepatitis B virus; MHN, massive hepatic necrosis; SMHN, submassive hepatic necrosis.

Aetiologies determine the pathogenesis of ALF, with viral infection, drug and toxin accounting for the three dominant aetiologies.^1 6 10^ Hepatotropic viral infections—hepatitis A virus (HAV), hepatitis E virus (HEV) and hepatitis B virus (HBV) (with or without hepatitis D virus (HDV)) – are the leading cause of ALF in developing countries. Notably, HCV rarely causes ALF.^10^ ALF can also occur in patients with autoimmune hepatitis (AIH).^10^ Among these aetiologies, MHN or SMHN is predominantly observed in patients with ALF associated with HAV, HBV, HEV, idiosyncratic drug reactions or a subset of AIH cases.^10^ In this review, the authors focus on the pathophysiology of MHN-associated ALF.

## Evolution of the nomenclature of ALF

When O’Grady and colleagues coined the definition of ALF, two seminal studies were specifically referenced. The first study was conducted in 1946.^12^ In this study, Lucke and Mallory systematically scrutinised ‘two frequently fatal types of acute hepatitis: a fulminant form with an extremely rapid outcome and a subacute form with a slower course’.^2 12^ Their detailed observations laid a solid histological foundation for future ALF research.^10^ The second pivotal study was published in 1970. Trey and Davidson introduced fulminant hepatic failure to describe a ‘potentially reversible condition, the consequence of severe liver injury, with an onset of encephalopathy within 8 weeks of the appearance of the first symptoms and in the absence of pre-existing liver disease’.^13^ This was the first formal definition of ALF.

The histological contribution of Lucke and Mallory remains worth emphasising even now, close to 80 years later. It is generally acknowledged that the most significant study on the pathological features of MHN-associated ALF was conducted in 1946.^12^ This groundbreaking study emerged during the Second World War.^12^ Lucke and Mallory analysed autopsy specimens from 178 patients, who were US Army personnel and died in an outbreak of ‘fatal hepatitis’. Among these patients, 53% were autopsied within 10 days of disease onset.^12^ We previously summarised the histological alterations of MHN based on Lucke and Mallory’s observations.^14^ Here, we revisit these findings because their deep insights continue to guide investigations into the mechanisms underlying MHN and liver regeneration. The key observations were as follows:

1. *Uniform hepatic involvement*: lesion affected all parts of the liver uniformly. While tissue destruction was often extreme and complete, residual hepatocytes occasionally persisted at the lobular periphery, forming a narrow rim.
2. *Progression of destruction*: the destruction extended from terminal hepatic veins to the periphery of lobes.
3. *Rapid clearance of dead cells*: dead hepatocytes were removed too rapidly for the earliest stages of disintegration to be observed. Even in rapidly fatal cases, no remnants of dead cells were detectable.
4. *Selective destruction of liver cells*: hepatocytes were specifically targeted, while the framework and sinusoids remained intact.
5. *Absence of scarring*: necrotic areas showed no evidence of scar formation.
6. *Inflammatory response*: a marked inflammatory reaction accompanied the destructive process, with inflammatory cellular infiltration being more prominent in the acute stage (<10 days) than in the subacute stage (>10 days). Macrophages/monocytes dominated the infiltrate, and inflammation was most conspicuous at the lobular periphery.
7. *Early ductular reaction (DR)*: cholangiocyte-mediated DR and regeneration commenced at an extremely early phase of destruction and persisted for a long time.
8. *Less common features*: endophlebitis of the efferent veins and jaundice were infrequent in patients with a clinical duration of less than 10 days.^12^,^14^

These prominent pathologic features of MHN raise a series of questions: (1) How does MHN occur? (2) Why are only hepatocytes targeted in MHN? (3) What is the nature of hepatocyte death? (4) Why and how are dead hepatocytes removed so rapidly? (5) Why do such extensive hepatocyte death and severe inflammation not induce a scar? (6) How can the severely damaged liver maintain liver function after MHN? (7) Why and how can the smallest cholangiocytes proliferate in such harsh conditions and mediate liver regeneration? (8) How can a liver suffering from MHN restore its damaged mass? Below, we discuss the state of the art on these issues.

## How does MHN occur?

MHN is the most severe liver injury within the spectrum of ALF. The occurrence of MHN depends on the aetiology. Only acute HAV, HBV and HEV infections, AIH or idiosyncratic drug hepatotoxicity induce MHN.^10^ Why and how MHN-associated ALF occurs in patients with associated aetiology remains largely unknown to date. However, some excellent studies have shed light on this issue—the mechanisms underlying MHN-associated ALF. For example, Farci and colleagues conducted a series of studies to investigate MHN in HBV-associated ALF over a decade.^15^,^18^ In 2010, Farci and colleagues systematically scrutinised two patients with HBV-associated ALF. Both underwent liver transplantation 8 days after symptom onset.^15^ Histological examination showed that the first patient suffered from MHN, characterised by no viable hepatocytes in the liver, whereas the second patient presented with SMHN. Both livers demonstrated extensive lymphoid cell infiltration. Microarray and immunohistochemistry analyses revealed the predominant B cell gene signature and the expression of B cell and plasma cell specific proteins, including CD20, CD138, immunoglobulin (Ig) G and IgM in both livers, suggesting an overwhelming B cell response centred in the liver with massive accumulation of plasma cells secreting IgG and IgM. Complement deposition was also observed in the livers of both patients. Remarkably, the researchers generated phage display Fab libraries of both IgG and IgM from the two patients to identify the targets of the antibodies produced in large quantities in the damaged livers. The hepatitis B core antigen (HBcAg) was confirmed as the molecular target of these antibodies. These antibodies displayed a restricted variable heavy chain (VH) repertoire and lacked somatic mutations. The two patients with unrelated ALF used an identical predominant VH gene with unmutated variable domain (*IGHV1-3*) for both IgG and IgM anti-HBc antibodies, indicating that HBcAg is the target of a germline human VH gene.^15^ Later, antibody binding to HBcAg was further confirmed by cryoelectron microscopy.^16^ These studies highlight the critical role of humoral immunity in the pathogenesis of MHN in HBV-associated ALF and are particularly noteworthy.

MHN is also observed in a subset of patients with AIH-induced ALF. Histologically, four criteria have been proposed to diagnose AIH-induced ALF, including the presence of MHN with prominent centrilobular haemorrhagic necrosis or the presence of MHN with persistent features of portal/periportal interface hepatitis.^19^ AIH-induced ALF usually occurs in patients with chronic AIH. Plasma cell infiltration in periportal and portal regions is a histological hallmark of AIH.^20^ The role of plasma cells in AIH-induced MHN has not yet been scrutinised, but warrants further study.

Notably, AIH-induced ALF does not occur in healthy individuals, but develops in patients with AIH.^10^ The similar phenomenon is also observed in HBV-associated ALF.^9^ ALF occurs not only in patients with acute HBV infection, but also in patients chronically infected with HBV when viral status changes.^9^ The nature and pathogenesis of MHN occurring in either a healthy liver or a cirrhotic liver are the same.^21^ However, ALF developing in individuals with chronic liver diseases conflicts with the current ALF definition, in which the absence of prior liver disease is a key criterion.^15^,^9^ In addition, acute-on-chronic liver disease (ACLF), a similarly urgent clinical syndrome that develops in cirrhotic patients with alcoholism or viral hepatitis infections (eg, HBV or HCV), has been recognised.^22^,^25^ Some experts suggest classifying patients with ALF and AIH or chronic HBV infection as ACLF.^10^ Given the controversial ACLF definition, we continue to categorise AIH-induced and HBV-induced MHN as ALF, regardless of the presence or absence of pre-existing liver disease.

## What is the nature of hepatocyte death?

Hepatocyte death can occur by different mechanisms depending on disease conditions, including necrosis, apoptosis, necroptosis, autophagy, pyroptosis and ferroptosis.^26^ According to modern definitions of cell death, the term ‘massive hepatic necrosis’ is misleading, as the nature of hepatocyte death in MHN is not exclusively necrosis. The term ‘massive necrosis’ was first used in 1939 to describe severe liver injury in rats fed with a vitamin B6-deficient diet.^27^ At that time, the concept of apoptosis and other cell death mechanisms had not yet been proposed. The Lucke and Mallory paper described virtually every pathologic feature of MHN.^10^ Based on their observations, Lefkowitch concluded that ‘the severe form of confluent necrosis in which the large majority of hepatocytes have undergone cell death by either necrosis, apoptosis or a combination of both (necroptosis)’.^10^ Our previous study demonstrated hepatocyte apoptosis characterised by cleaved caspase-3-positive cells in MHN.^28^ However, few studies have confirmed necroptosis in patients with MHN-associated ALF to date.

An interesting and important issue is whether the degree of hepatocyte death is associated with the clinical outcome of patients with ALF. Based on a study in 1975,^29^ a recent review states that ‘the degree of necrosis has been identified in several studies as related to outcome, with >50%–70% necrosis identified as the tipping point for poor outcomes’.^6^ This conclusion is controversial because two clinical studies performed in the 1990s showed that the clinical outcome of patients with ALF, whether with previously healthy livers or cirrhotic livers, does not correlate with the extent of necrosis.^30 31^ To clarify this issue, comprehensive studies that fully consider aetiologies, clinical course and underlying comorbidities are needed in the future.

## How can the severely damaged liver maintain liver function after MHN?

Following MHN, how does a severely damaged liver still perform essential liver functions to support systemic homeostasis? MHN does not immediately result in death in the majority of patients with ALF because the remaining hepatocytes and activated liver progenitor cells (LPCs) retain the capacity to perform fundamental liver functions, sustaining these patients’ lives.^32^ Given the severely reduced numbers and functions of leftover hepatocytes, the efficacy of activated LPCs determines whether apatient with ALF will survive the golden window—the first 7 days after the disease onset.^32^ Therefore, LPC proliferation and performance of hepatic functions are key events determining the clinical outcome of apatient with MHN-associated ALF.

Prior to discussing LPC biology in MNH-associated ALF, it is essential to first clarify a critical question: what is a LPC? To date, a large number of LPC-relevant studies have been published. In 2004, a common nomenclature for LPC was established by an expert group.^33^ This expert consensus clearly defined several vital structures of normal biliary tree, which are essential for understanding LPC biology. The canal of Hering was described as the ‘physiologic link between hepatocyte canaliculi and the biliary tree; partially lined by hepatocytes and by cholangiocytes (not by cells of intermediate morphology, which are not identified in normal livers)’. Bile ductules were depicted as a ‘Link between canals of Hering and the interlobular bile ducts; lined entirely by cholangiocytes, may begin at the edge of portal tract stroma or may traverse the limiting plate, in which case it will have an ‘intralobular’ as well as an ‘intraportal’ segment.’ ‘Isolated’ cholangiocytes or progenitor cells in two-dimensional tissue sections were defined as, ‘These cells are often, if not always, cross sections of canals of Hering and intralobular bile ductules and, therefore, not necessarily isolated. They may be referred to by the immunomarkers used to define them (eg, cytokeratin (CK) 19^+^, CK7^+^, neural cell adhesion molecule (NCAM^+^)) and by the function which is under investigation (eg, ‘cholangiocytes’, ‘progenitor cells’), always recognising, however, that they have multiple functions’. DR refers to ‘A reaction of ductular phenotype, possibly but not necessarily of ductular origin, in acute and chronic liver disease’. It may arise from: (1) proliferation of pre-existing cholangiocytes; (2) progenitor cells (local and/or circulating cells probably bone marrow-derived); (3) rarely, biliary metaplasia of hepatocytes.’ Intermediate hepatobiliary cells are ‘cells in ductular reactions that are morphologically or phenotypically intermediate between hepatocytes and cholangiocytes: >6 microns, <40 microns, dual phenotyping by immunostaining or by electron microscopy’. After more than two decades, these terminologies have stood the test of time and remain particularly critical to understanding the pathophysiology of ALF with MHN.

The LPC is currently a hot research topic in hepatology and stem cell biology. However, the existence and definition of LPCs have been controversial in the scientific community. It is easy to consider LPCs as a population of naïve adult stem cells in the human liver. This, however, is not true. LPCs belong to a tissue stem cell category. In humans, there is a fundamental covenant among cells: (1) somatic cells stop reproduction and perform unique functions; (2) germ cells propagate the genes; (3) stem cells repair tissues.^34^ Some organs (eg, skin and intestine) possess unique organ-specific stem cells, whereas the liver does not.^34^ However, this does not mean that the liver lacks stem-like cells to perform repairs in cases of severe liver diseases. Since Shinya Yamanaka and colleagues discovered that mature cells can be reprogrammed to become pluripotent, a clear boundary between somatic cells and stem cells has ceased to exist.^35^ When provided with the necessary transcription factors under special conditions (eg, forced transcription factor expression in cultured somatic cells), different cells can reprogram into each other through differentiation, transdifferentiation and rejuvenation.^36^ However, somatic cells cannot reprogram spontaneously in vivo due to physiological and pathophysiological limitations.

In adult livers, both hepatocytes and cholangiocytes can transdifferentiate into each other depending on disease conditions and repair demands.^37^ In most studies, liver cells simultaneously expressing both hepatocyte markers (eg, hepatocyte nuclear factor 4α (HNF4α), HNF1α, CK8 and CK18) and cholangiocyte markers (SRY-box transcription factor (SOX)9, CK7 and CK19) are termed LPCs. Based on this terminology, LPCs represent a transient intermediate cell state in liver injury, not a cell type present in the homeostatic liver. Such cells are rarely observed in a normal liver. However, in various liver diseases, hepatocytes may express biliary transcription factors (eg, SOX9) and biliary keratin (CK7 and CK19), while the smallest cholangiocytes residing in ductules and canals of Hering can express hepatocyte transcription factors (eg, HNF4α and HNF1α) and hepatic keratin (CK8 and CK18). The former is often observed in cholestasis. A classic example is primary biliary cholangitis (PBC). In longstanding PBC-dependent cholestasis, the neoductules, lined by cuboidal or flattened cells, are thought to arise from dedifferentiation or metaplasia of periportal hepatocytes, and to a lesser extent, from proliferation of pre-existing ductules, depending on the degree of biliary obstruction (see the patient with PBC presented in Figure 10.20 of the textbook).^11^ Here, periportal hepatocytes act as LPCs by co-expressing biliary markers (CK7 and CK19) to repair damaged bile ducts. Similar phenomena have also been demonstrated in experimental animals fed with 3,5-diethoxycarbonyl-1,4-dihydrocollidine (DDC) or choline-deficient ethionine-supplemented (CDE) diets.^38^,^40^

In contrast to hepatocyte dedifferentiation in cholestasis, the smallest cholangiocytes expressing hepatocyte phenotypes are commonly observed in patients with MHN-associated ALF.^33 37^ In extremely severe MHN cases, no hepatocytes remain in patients with ALF (see patient 3^12^ and patient 1).^41^ In such a case, dedifferentiation of mature hepatocytes is not possible since hepatocytes no longer exist. What about patients with MHN-associated ALF retaining 30% of hepatocytes? Do these remaining hepatocytes dedifferentiate into LPCs or LPC-like cells? We propose the following explanations: (1) In the liver, hepatocytes are the primary cells performing functions essential for systemic homeostasis, while other liver cells act as supportive cells that enable hepatocyte function.^42^ Following MHN, the remaining fewer than 30% of hepatocytes are insufficient to produce sufficient quantities of essential liver proteins. In this extreme scenario, the optimal strategy for patient survival is to let remaining hepatocytes produce liver function proteins maximally, while activating the second pathway of liver regeneration (LPC activation) to compensate for hepatocyte functions. (2) Theoretically, remaining hepatocytes could increase their numbers through proliferation. However, hepatocyte proliferation in MHN-associated ALF does not occur. In a recent study, we found that high levels of hepatocyte growth factor (HGF)-phosphorylated MET (p-MET)-phosphorylated signal transducer and activator of transcription (STAT)3 and epidermal growth factor (EGF)-phosphorylated epidermal growth factor receptor (p-EGFR)-phosphorylated extracellular signal-regulated kinase (p-ERK) signalling are active only in activated LPCs, not in remaining hepatocytes. These results indicate that remaining hepatocytes lack sensitivity to growth factor stimulation in this context.^43^ (3) Cell differentiation and reprogramming cannot be finalised in one cell cycle.^44^ Ng and Gurdon reported that cell memory persists through 24 cell cycles without transcription, indicating that lineage change requires multiple cell cycles.^45^ In MHN-associated ALF, LPC-derived intermediate hepatocytes appear approximately 3 weeks after disease onset.^14 32^ Therefore, proliferation is a prerequisite for cell differentiation. As mentioned, remaining hepatocytes do not proliferate in MHN-associated ALF. This explains their inability to initiate dedifferentiation in this harsh disease environment.

Collectively, LPC activation, hepatocyte function performance and differentiation into mature hepatocytes are critical to rescue patients’ lives and recover liver architecture/function in MHN-associated ALF. Mature hepatocyte dedifferentiation is therefore not viable. Therefore, the 2004 nomenclature clearly highlights that LPCs are smallest cholangiocytes residing in the canal of Hering and the smallest biliary tree branches.^33 46^ In the setting of MHN, the majority of hepatocytes are destroyed. However, the massive loss of hepatocytes does not immediately kill patients with ALF, indicating that these severely damaged livers still maintain essential liver functions to support systemic homeostasis. Vital liver function proteins are mainly produced by hepatocytes. However, the number and function of residual hepatocytes are severely compromised after MHN. At this critical moment, LPCs replace hepatocytes to perform liver functions. Lucke and Mallory’s study, along with subsequent numerous clinicopathological studies, show that the smallest cholangiocyte-mediated DR and regeneration begin at an extremely early phase of destruction and persist for a long time.^12 14 28^ It has been well recognised that LPCs express master hepatic transcription factors (eg, HNF4α and C/EBPα) and vital liver function proteins (eg, albumin, coagulation factors and carbamoyl phosphate synthetase I (CPS1)) in patients with MHN.^47^,^49^ Over time, intermediate hepatobiliary cells are observed in the regenerated liver. These clinical data provide evidence that LPCs are indeed identical to the smallest cholangiocytes.

Complementing these clinical observations, zebrafish provide an animal model demonstrating how cholangiocytes mediate liver regeneration when nearly all hepatocytes are destroyed.^50^ In 2014, two independent studies used metronidazole (MTZ) to kill nearly all hepatocytes in larval and adult zebrafish; and subsequently, they washed out the toxin.All zebrafish treated with MTZ rapidly restored the liver mass through cholangiocyte proliferation and transdifferentiation into hepatocytes.^51 52^ In an editorial evaluating these two elegant studies, Catherine Verfaillie referred to the studies as ‘Biliary Cells to the Rescue of Prometheus’.^50^

**Figure 2:**
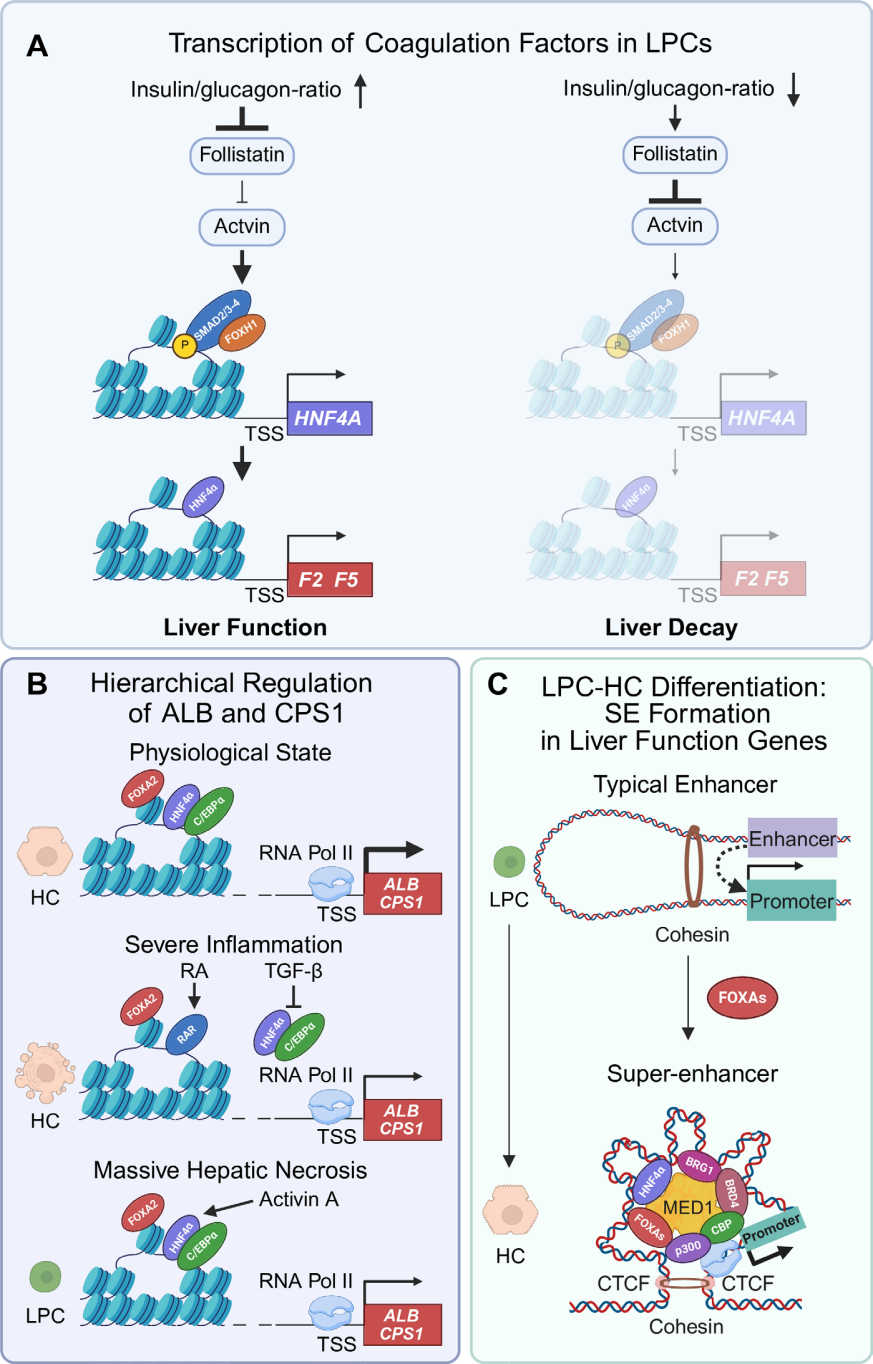
Mechanisms by which LPCs perform hepatocyte functions and differentiate into hepatocytes. (**A**) Following MHN, the expression of coagulation factors in LPCs is activated by activin. Activin signalling in LPCs is negatively regulated by follistatin, a hormone controlled by insulin and glucagon. (**B**) A hepatic hierarchical transcription network regulates the transcription of *ALB* and *CPS1*. Under physiological conditions, hepatocytes use the pioneer factor FOXA2 to support the master transcription factors HNF4α and C/EBPα in regulating the transcription of these two genes. In cases of severe liver injury, both HNF4α and C/EBPα are inhibited by inflammatory cytokines such as TGF-β. FOXA2 then coordinates with the alternative transcription factor RAR to maintain *ALB* and *CPS1* transcription in hepatocytes. When the liver suffers from MHN, activated LPCs express the three key transcription factors—FOXA2, HNF4α and C/EBPα—to initiate the transcription of *ALB* and *CPS1*. (**C**) Liver function genes are associated with super-enhancers in hepatocytes, but not in LPCs. During the differentiation of LPCs into hepatocytes, LPCs form super-enhancers on liver function genes, supported by FOXAs. Created with BioRender.com. ALB, albumin; BRD4, bromodomain-containing protein 4; BRG1, brahma-related gene 1; C/EBP, CCAAT/enhancer binding protein; CPS1, carbamoyl phosphate synthetase I; CTCF, CCCTC-binding factor; FOXA, forkhead box A; FOXH1, forkhead box H1; HC, hepatocyte; HNF4α, hepatocyte nuclear factor 4α; LPC, liver progenitor cell; MED1, mediator complex subunit 1; MHN, massive hepatic necrosis; RA, retinoic acid; RAR, retinoic acid receptor; SE, super-enhancer; SMAD, SMA and MAD-related protein; TGF-β, transforming growth factor-β; TSS, transcription start site.

To date, the detailed mechanisms underlying LPC activation, rapid proliferation and execution of hepatic functions remain largely unknown. Whether LPCs adequately perform liver functions is the key determinant of the clinical outcome of patients with MHN-associated ALF. Recently, the regulatory effects of activin, a member of the transforming growth factor (TGF-β) superfamily, in enabling LPCs to perform coagulation function in ALF was reported. When patients with ALF suffer from MHN, activin, which is derived from inflammatory cells and dead hepatocytes, stimulates LPCs to express the master hepatic transcription factor HNF4α via the SMA and MAD-related protein (SMAD)-Forkhead Box H1 (FOXH1) complex. Subsequently, HNF4α activates the transcription of coagulation factors in LPCs (figure 2A). Thus, activated LPCs rescue coagulation function in ALF.^47^ To date, liver transplantation remains the only curative approach for patients with ALF. In clinical practice, enrolment of patients with severe liver diseases onto the waiting list for liver transplantation is determined by the Model for End-stage Liver Disease (MELD) scores.^53^ The MELD score incorporates three variables: INR, serum bilirubin and serum creatinine (MELD=3.78× ln [serum bilirubin (mg/dL)] + 11.2× ln (INR) + 9.57× ln [serum creatinine (mg/dL)] + 6.43). INR contributes substantially to the MELD equation to gauge disease severity in patients with ALF, highlighting the critical role of coagulation in the survival of patients with ALF. The essential regulatory effect of activin on the expression of coagulation factors in LPCs may offer a novel therapeutic strategy to improve clinical outcomes in patients with MHN-associated ALF.

Under MHN conditions, the regenerative response associated with MHN is a division of labour shared between residual hepatocytes and LPCs.^10^ A key question is how leftover hepatocytes synergise with LPCs to maintain liver functions. Recent studies depict a hierarchical regulatory network that maintains homeostasis of albumin and urea cycle in different scenarios.^48 54^ These studies investigated the role of two master hepatic transcription factors, HNF4α and C/EBPα, in regulating vital hepatocyte functions such as the expression of albumin and the urea cycle rate-limiting enzyme CPS1. In normal hepatocytes, the expression of HNF4α and C/EBPα requires the pioneer factor FOXA2. Critically, FOXA2 is indispensable for HNF4α-dependent or C/EBPα-dependent transcription of key hepatocyte function genes (eg, *ALB* and *CPS1*), as it opens chromatin and maintains accessibility at these loci. In collaboration with FOXA2, HNF4α and C/EBPα regulate these vital hepatic genes under physiological conditions. When HNF4α and C/EBPα expression is inhibited by inflammatory cytokines such as TGF-β, the alternative transcription factor retinoid acid receptor (RAR) is activated by its ligand retinoid acid and replaces HNF4α and C/EBPα to activate target gene transcription. RAR activity also depends on FOXA2 to maintain chromatin accessibility. In patients with MHN, FOXA2 synergises with HNF4α and C/EBPα to mediate transcription of hepatocyte function genes in LPCs (figure 2B). Whether such a hierarchical regulatory network represents a universal paradigm for the liver’s response to different microenvironmental challenges warrants future investigation.

How do LPCs rapidly acquire hepatocytic functions during MHN? Our recent study showed that the formation of super-enhancers at liver function genes is essential for LPCs to adequately express hepatocyte identity genes.^55^ Super-enhancers are putative enhancer clusters with unusually high levels of enhancer activity, which are occupied by transcription factors, cofactors, chromatin regulators and core transcription apparatus, including master transcription factors (eg, octamer-binding transcription factor 4 (OCT4), SOX2, homeobox protein NANOG), the mediators, coactivators, insulators (CTCF and cohesin), chromatin regulators (structural maintenance of chromosomes 1 (SMC1), Brahma-related gene 1 (Brg1)) and the histone acetyltransferase CREB-binding protein (CPB)/E1A binding protein p300.^56^ Super-enhancers play critical roles in controlling cell state and identity.^56^

We found that a large number of liver function-associated genes in hepatocytes form super-enhancers, essential epigenetic structures for performing hepatocyte functions.^55^ In contrast, quiescent LPCs lack super-enhancers at the respective sites. However, super-enhancers are formed during differentiation into hepatocytes. Members of the pioneer factor FOXA family, particularly FOXA2, are key regulators of super-enhancer formation and maintenance at these loci^55^ (figure 2C).

## How can the smallest cholangiocytes proliferate in such harsh conditions and mediate liver regeneration?

Recently, we conducted a study investigating the potential signalling networks that rapidly trigger LPC proliferation and initiate hepatocyte gene expression.^43^ Spatial transcriptomics analysis based on liver tissues from four patients with MHN-associated ALF identified multiple signals received by LPCs, including macrophage-derived TGF-β and hepatic stellate cell-derived HGF, as well as EGF produced by both cell types.^43^ TGF-β plays fundamental roles in multiple biological processes, including development, adult tissue homeostasis and regeneration.^57^ TGF-β signalling is regarded as a guardian in the preservation of systemic, tissue and cell integrity.^57^ TGF-β acts as a key factor that governs proliferation in many types of normal cells.^57^ Weinberg thought that TGF-β has the last word in determining whether these cells proliferate.^58^ In LPCs, TGF-β inhibits cell proliferation through impeding G1-S phase transition.^43^ To control the LPC cell cycle, the most important TGF-β signalling targets are the genes *CDKN1A* and *CDKN2B*, which encode the two CDK inhibitors p15^INK4B^ and p21^Cip1^, respectively.^43^ However, in patients with ALF, robust TGF-β-p-SMAD2 signalling did not suppress LPC proliferation.^43^ Immunohistochemistry revealed remarkably increased expression of p-STAT3 and p-ERK in LPCs, indicating active HGF and EGF signalling within these cells.^43^ We further found that both HGF and EGF promoted LPC proliferation even in the presence of TGF-β. Beyond acting as mitogens, EGF and HGF regulated master cholangiocyte genes (eg, *SOX9*) and hepatocyte genes (eg, *HNF4A*), respectively, in LPCs.^43^ Notably, TGF-β-activated SMADs are essential for the transcription of EGF-induced *SOX9* and HGF-dependent *HNF4A*.^43^ This preliminary study provides evidence highlighting that microenvironmental signalling networks govern LPC proliferation and hepatocyte gene expression in patients with MHN-associated ALF. The detailed cellular and molecular mechanisms require further investigation.

## How can a liver suffering from MHN restore its damaged mass?

We have described how LPCs rapidly proliferate and perform vital liver functions during and after MHN. Over time, these regenerated LPCs differentiate into hepatocytes, thereby restoring parenchymal mass. To date, how LPCs differentiate into hepatocytes after MHN remains largely unknown. As MHN-associated ALF is a severe clinical syndrome, patients rarely tolerate liver tissue sampling. In most cases, histological examinations are performed on explants following liver transplantation. Dynamic histological observations in patients with MHN-associated ALF are rare. To date, molecular mechanisms underlying LPC-to-hepatocyte differentiation are primarily derived from experimental studies, particularly from the zebrafish model.

ALF models established in rodents (eg, mice treated with acetaminophen) do not develop MHN, as high concentrations of toxins rapidly induce lethal toxicity.^32^ In contrast to rodents, zebrafish demonstrate remarkable regenerative capacity even when hepatocytes are completely destroyed.^51 52^ In 2014, He *et al* and Choi *et al* developed transgenic fish larvae and adult fish by incorporating bacterial nitroreductase (NTR) downstream of hepatic specific promoters: the liver-type fatty acid binding protein (*lfabp*) or fatty acid binding protein-10a promoters. The models were designed as *Tg(lfabp:mCherry-NTR*) and *Tg(fabp10a:CFP-NTR*), respectively.^51 52^ Following the administration of MTZ, nearly all hepatocytes were eliminated. Upon MTZ withdrawal, biliary cells rapidly transdifferentiate into hepatocytes, leading to the restoration of parenchymal mass.^51 52^ Unlike in mammals, where the smallest cholangiocytes mediate post-MHN regeneration, nearly all biliary cells contribute to this process in zebrafish.^51 52^ This discrepancy reflects evolutionary divergence in the biliary system between fish and mammals.

Based on this zebrafish model, Shin and colleagues have conducted a series of studies investigating how LPC/cholangiocytes differentiate into hepatocytes after complete loss of hepatocytes.^59^,^64^ They found that LPC-to-hepatocyte differentiation is regulated by multiple signalling pathways, including BMP, STAT3-SOCS3, Notch-SOX9, PI3K-AKT-mTOR and EGFR-ERK-SOX9.^59^,^64^ In addition, bromodomain and extra-terminal (BET) proteins and histone deacetylase 1 (HDAC1) also play critical roles in LPC-to-hepatocyte differentiation following hepatocyte loss in zebrafish.^61 65^ BET proteins contribute to regulating the dedifferentiation of cholangiocytes, proliferation of intermediate hepatocytes and maturation of cholangiocyte-derived hepatocytes.^65^ HDAC1 regulates LPC-to-hepatocyte differentiation by inhibiting *Sox9b*.^61^ Furthermore, a recent study demonstrated that interfering with VEGFR influences differentiation from LPCs to hepatocytes.^66^ These findings provide critical insights for the future investigation to clarify how patients with ALF restore liver mass following MHN.

## Summary

MHN-associated ALF represents the most severe form of liver injury in human diseases and has unique aetiologies (eg, HAV, HBV and HEV, as well as AIH). In this critical scenario, LPCs play leading roles in maintaining vital liver functions and mediating liver regeneration to restore lost parenchymal mass. Future studies on LPC biology should seek detailed answers to three main questions:

1. How does a severely damaged liver initiate LPC proliferation following MHN?
2. How do LPCs take over hepatocyte functions to maintain essential liver functions?
3. How do LPCs adequately differentiate into hepatocytes?

**Figure 3:**
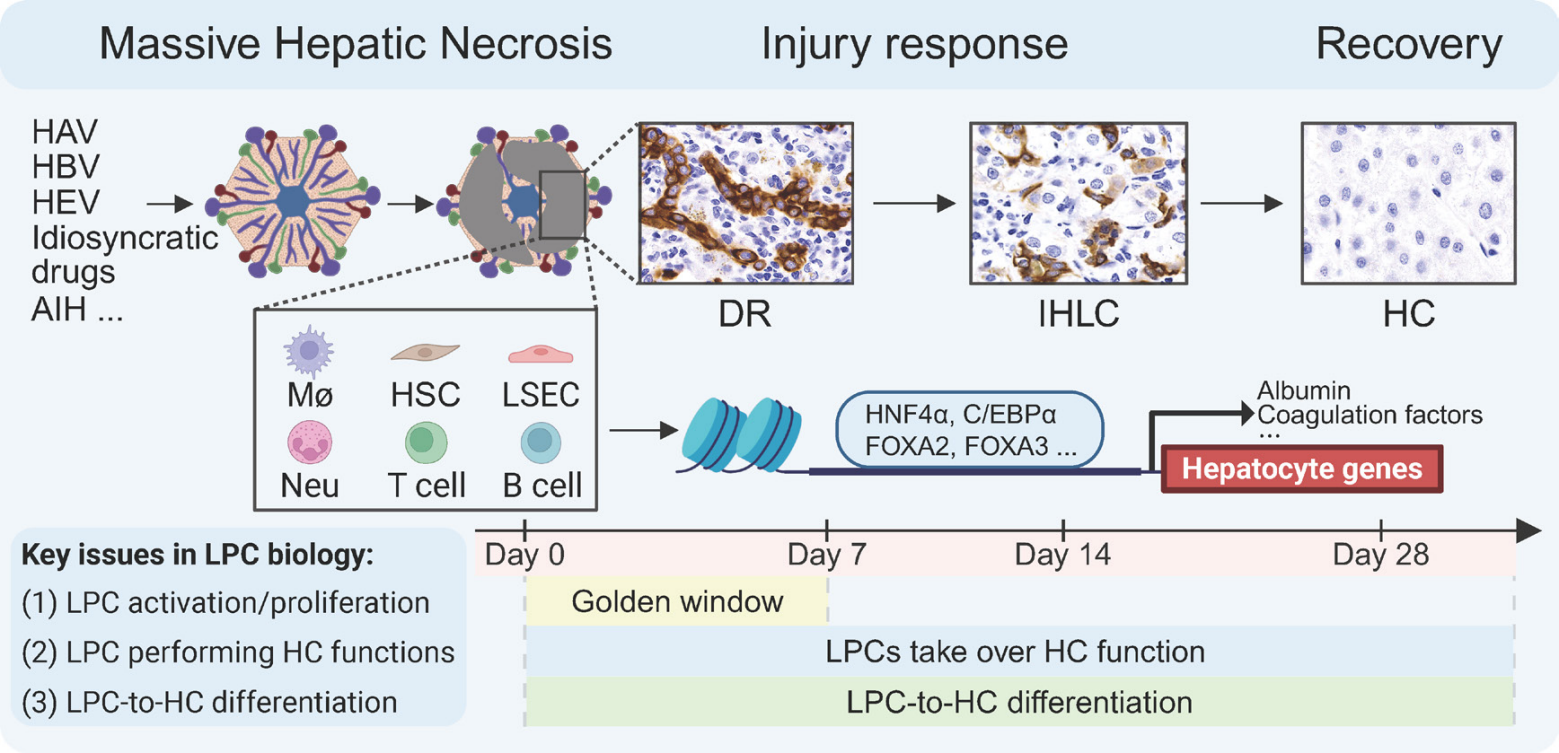
The scheme summarises critical aspects of MHN-associated ALF. MHN-associated ALF has unique aetiologies, including HAV, HBV, HEV, AIH and idiosyncratic drugs. Following MHN, HSCs, Mϕs, LSECs, other inflammatory cells (eg, neutrophils, NK cells, T cells and B cells) are activated or recruited, thereby constituting disease microenvironment. One week after disease onset is a ‘golden window’. LPCs are rapidly activated and proliferate following MHN. Whether LPCs rapidly and sufficiently take over hepatocyte functions determines the performance during the golden window and subsequent clinical outcome. Over time, LPCs differentiate into mature hepatocytes, thereby recovering damaged liver architecture. Hepatic master transcription factors (HNF4α, C/EBPα, FOXA2 and FOXA3) play critical roles in LPC activation and transdifferentiation into mature hepatocytes. Created with BioRender.com. AIH, autoimmune hepatitis; ALF, acute liver failure; C/EBP, CCAAT/enhancer binding pEnhancer Binding Protein; DR, ductular reaction; FOXA, forkhead bForkhead Box A; HAV, hepatitis A virus; HBV, hepatitis B virus; HC, hepatocyte; HEV, hepatitis E virus; HNF4α, hepatocyte nuclear faHepatocyte Nuclear Factor 4α; HSCs, hepatic stellate cells; IHLC, intermediate hepatocyte-like cells; LPCs, liver progenitor cells; LSECs, liver sinusoidal endothelial cells; MHN, massive hepatic necrosis; Mϕs, macrophages; Neu, neutrophils; NK, natural killer.

The behaviour of LPCs activated by MHN is modulated by the disease microenvironment. The interaction between activated LPCs and surrounding cells—including activated hepatic stellate cells, macrophages and other inflammatory cells—is crucial for understanding LPC-mediated liver regeneration post MHN (figure 3). Given the rapid progress of MHN, there is almost no chance for clinicians to observe the complete sequence of liver damage and associated systemic changes. Furthermore, the lack of reliable rodent models remains a major bottleneck in elucidating MHN-associated ALF mechanisms. While zebrafish elegantly model cholangiocytes/LPCs-mediated liver regeneration after nearly total hepatocyte loss, the evolutionary divergence between fish and mammals (eg, absence of Kupffer cells in zebrafish^67^) must be carefully considered.

As you have noticed by now, we do not address three of the initial eight key questions: (2) ‘Why are only hepatocytes targeted in MHN?’ (4) ‘Why and how are dead hepatocytes removed so rapidly?’ and (5) ‘Why do such extensive hepatocyte death and severe inflammation not induce a scar?’. The reason for this omission is that to date, no convincing studies are available to address these critical issues. At least two key challenges impede progress in resolving them. First, MHN-associated ALF develops extremely rapidly. In clinical practice, clinicians rarely have the opportunity to observe and examine a patient during the active phase of MHN. Second, MHN-associated ALF has a unique, aetiology-dependent nature, particularly when caused by HAV, HBV and HEV. To date, there is no ideal model that accurately mimics MHN-associated ALF. As mentioned in this review, zebrafish provide a valuable model to study cholangiocyte-mediated liver regeneration following complete hepatocyte ablation. However, because this model relies on genetically modified fish, the mechanisms of cell death in zebrafish differ from those in human patients.

## References

[1] Maiwall R, Kulkarni AV, Arab JP (2024). Acute liver failure. Lancet.

[2] Williams R, Schalm SW, O’Grady JG (1993). Acute liver failure: redefining the syndromes. Lancet.

[3] Lee WM, Stravitz TR, Larson AM (2012). Introduction to the revised American Association for the Study of Liver Diseases position paper on acute liver failure 2011. Hepatology.

[4] European Association for the Study of the Liver. Electronic address: easloffice@easloffice.eu; Clinical practice guidelines panel, Wendon, J, *et al* (2017). EASL Clinical Practical Guidelines on the management of acute (fulminant) liver failure. J Hepatol.

[5] Bernal W, Lee WM, Wendon J (2015). Acute liver failure: A curable disease by 2024?. J Hepatol.

[6] Stravitz RT, Fontana RJ, Karvellas C (2023). Future directions in acute liver failure. Hepatology.

[7] Stravitz RT, Lee WM (2019). Acute liver failure. Lancet.

[8] Bernal W, Wendon J (2013). Acute liver failure. N Engl J Med.

[9] Bernal W, Auzinger G, Dhawan A (2010). Acute liver failure. Lancet.

[10] Lefkowitch JH (2016). The Pathology of Acute Liver Failure. Adv Anat Pathol.

[11] Alastair D, Burt LDF, Hübscher SG (2012). MacSween pathology of the liver.

[12] Lucké B, Mallory T (1946). The Fulminant Form of Epidemic Hepatitis. Am J Pathol.

[13] Trey C, Davidson CS (1970). The management of fulminant hepatic failure. Prog Liver Dis.

[14] Weng H-L, Cai X, Yuan X (2015). Two sides of one coin: massive hepatic necrosis and progenitor cell-mediated regeneration in acute liver failure. Front Physiol.

[15] Farci P, Diaz G, Chen Z (2010). B cell gene signature with massive intrahepatic production of antibodies to hepatitis B core antigen in hepatitis B virus-associated acute liver failure. Proc Natl Acad Sci U S A.

[16] Wu W, Chen Z, Cheng N (2013). Specificity of an anti-capsid antibody associated with Hepatitis B Virus-related acute liver failure. J Struct Biol.

[17] Chen Z, Diaz G, Pollicino T (2018). Role of humoral immunity against hepatitis B virus core antigen in the pathogenesis of acute liver failure. Proc Natl Acad Sci USA.

[18] Chen Z, Shen C, Engle RE (2020). Next‐generation sequencing of the intrahepatic antibody repertoire delineates a unique B‐cell response in HBV‐associated acute liver failure. J Viral Hepat.

[19] Stravitz RT, Lefkowitch JH, Fontana RJ (2011). Autoimmune acute liver failure: Proposed clinical and histological criteria. Hepatology.

[20] JH L (2016). Scheuer’s liver biopsy interpretation.

[21] POPPER H, ELIAS H (1955). Histogenesis of hepatic cirrhosis studied by the threedimensional approach. Am J Pathol.

[22] Yu X, Zhou R, Tan W (2024). Evidence-based incorporation of key parameters into MELD score for acute-on-chronic liver failure. eGastroenterology.

[23] Arroyo V, Moreau R, Jalan R (2020). Acute-on-Chronic Liver Failure. N Engl J Med.

[24] Moreau R, Jalan R, Gines P (2013). Acute-on-chronic liver failure is a distinct syndrome that develops in patients with acute decompensation of cirrhosis. Gastroenterology.

[25] Jalan R, Williams R (2002). Acute-on-chronic liver failure: pathophysiological basis of therapeutic options. Blood Purif.

[26] Cao P, Jaeschke H, Ni H-M (2025). The Ways to Die: Cell Death in Liver Pathophysiology. Semin Liver Dis.

[27] György P, Goldblatt H (1939). HEPATIC INJURY ON A NUTRITIONAL BASIS IN RATS. J Exp Med.

[28] Li H, Xia Q, Zeng B (2015). Submassive hepatic necrosis distinguishes HBV-associated acute on chronic liver failure from cirrhotic patients with acute decompensation. J Hepatol.

[29] Portmann B, Talbot IC, Day DW (1975). Histopathological changes in the liver following a paracetamol overdose: correlation with clinical and biochemical parameters. J Pathol.

[30] Chenard-Neu MP, Boudjema K, Bernuau J (1996). Auxiliary liver transplantation: regeneration of the native liver and outcome in 30 patients with fulminant hepatic failure--a multicenter European study. Hepatology.

[31] Hanau C, Munoz SJ, Rubin R (1995). Histopathological heterogeneity in fulminant hepatic failure. Hepatology.

[32] Lin T, Feng R, Liebe R (2022). Liver Progenitor Cells in Massive Hepatic Necrosis—How Can a Patient Survive Acute Liver Failure?. Biomolecules.

[33] Roskams TA, Theise ND, Balabaud C (2004). Nomenclature of the finer branches of the biliary tree: Canals, ductules, and ductular reactions in human livers. Hepatology.

[34] Stephen C, Stearns RM (2015). Evolutionary medicine.

[35] Takahashi K, Tanabe K, Ohnuki M (2007). Induction of pluripotent stem cells from adult human fibroblasts by defined factors. Cell.

[36] Takahashi K, Yamanaka S (2016). A decade of transcription factor-mediated reprogramming to pluripotency. Nat Rev Mol Cell Biol.

[37] Desmet VJ (2011). Ductal plates in hepatic ductular reactions. Hypothesis and implications. I. Types of ductular reaction reconsidered. Virchows Arch.

[38] Jörs S, Jeliazkova P, Ringelhan M (2015). Lineage fate of ductular reactions in liver injury and carcinogenesis. J Clin Invest.

[39] Wu B, Shentu X, Nan H (2024). A spatiotemporal atlas of cholestatic injury and repair in mice. Nat Genet.

[40] Li L, Cui L, Lin P (2023). Kupffer-cell-derived IL-6 is repurposed for hepatocyte dedifferentiation via activating progenitor genes from injury-specific enhancers. Cell Stem Cell.

[41] Farci P, Wollenberg K, Diaz G (2012). Profibrogenic chemokines and viral evolution predict rapid progression of hepatitis C to cirrhosis. Proc Natl Acad Sci U S A.

[42] Adler M, Chavan AR, Medzhitov R (2023). Tissue Biology: In Search of a New Paradigm. Annu Rev Cell Dev Biol.

[43] Tong C, Lin T, Wang H (2025). TGF-β serves as a critical signaling determinant of liver progenitor cell fate and function. bioRxiv.

[44] Bruce Alberts AJ, Lewis J, Raff M (2002). Molecular biology of the cell.

[45] Ng RK, Gurdon JB (2008). Epigenetic memory of an active gene state depends on histone H3.3 incorporation into chromatin in the absence of transcription. Nat Cell Biol.

[46] Roskams T (2008). Relationships Among Stellate Cell Activation, Progenitor Cells, and Hepatic Regeneration. Clin Liver Dis.

[47] Lin T, Wang S, Munker S (2022). Follistatin-controlled activin-HNF4α-coagulation factor axis in liver progenitor cells determines outcome of acute liver failure. Hepatology.

[48] Feng R, Kan K, Sticht C (2022). A hierarchical regulatory network ensures stable albumin transcription under various pathophysiological conditions. Hepatology.

[49] Feng R, Tong C, Lin T (2024). Insulin Determines Transforming Growth Factor β Effects on Hepatocyte Nuclear Factor 4α Transcription in Hepatocytes. Am J Pathol.

[50] Verfaillie CM (2014). Biliary cells to the rescue of Prometheus. Gastroenterology.

[51] He J, Lu H, Zou Q (2014). Regeneration of liver after extreme hepatocyte loss occurs mainly via biliary transdifferentiation in zebrafish. Gastroenterology.

[52] Choi T-Y, Ninov N, Stainier DYR (2014). Extensive conversion of hepatic biliary epithelial cells to hepatocytes after near total loss of hepatocytes in zebrafish. Gastroenterology.

[53] European Association for the Study of the Liver (2024). EASL Clinical Practice Guidelines on liver transplantation. J Hepatol.

[54] Feng R, Liu R, Tong C (2023). FOXA2 is essential for maintaining the urea cycle in acute liver failure. bioRxiv.

[55] Lin T, Huang C, Tong C (2025). Regulation of essential hepatocyte functions and identity by super-enhancers in health and disease. bioRxiv.

[56] Hnisz D, Abraham BJ, Lee TI (2013). Super-Enhancers in the Control of Cell Identity and Disease. Cell.

[57] Massagué J, Sheppard D (2023). TGF-β signaling in health and disease. Cell.

[58] W RA (2014). The biology of cancer.

[59] So J, Kim M, Lee S-H (2021). Attenuating the Epidermal Growth Factor Receptor-Extracellular Signal-Regulated Kinase-Sex-Determining Region Y-Box 9 Axis Promotes Liver Progenitor Cell-Mediated Liver Regeneration in Zebrafish. Hepatology.

[60] Jung K, Kim M, So J (2021). Farnesoid X Receptor Activation Impairs Liver Progenitor Cell-Mediated Liver Regeneration via the PTEN-PI3K-AKT-mTOR Axis in Zebrafish. Hepatology.

[61] Ko S, Russell JO, Tian J (2019). Hdac1 Regulates Differentiation of Bipotent Liver Progenitor Cells During Regeneration via Sox9b and Cdk8. Gastroenterology.

[62] Russell JO, Ko S, Monga SP (2019). Notch Inhibition Promotes Differentiation of Liver Progenitor Cells into Hepatocytes via *sox9b* Repression in Zebrafish. Stem Cells Int.

[63] Khaliq M, Ko S, Liu Y (2018). Stat3 Regulates Liver Progenitor Cell-Driven Liver Regeneration in Zebrafish. Gene Expr.

[64] Choi T, Khaliq M, Tsurusaki S (2017). Bone morphogenetic protein signaling governs biliary‐driven liver regeneration in zebrafish through tbx2b and id2a. Hepatology.

[65] Ko S, Choi T-Y, Russell JO (2016). Bromodomain and extraterminal (BET) proteins regulate biliary-driven liver regeneration. J Hepatol.

[66] Rizvi F, Lee Y-R, Diaz-Aragon R (2023). VEGFA mRNA-LNP promotes biliary epithelial cell-to-hepatocyte conversion in acute and chronic liver diseases and reverses steatosis and fibrosis. Cell Stem Cell.

[67] Goessling W, Sadler KC (2015). Zebrafish: an important tool for liver disease research. Gastroenterology.

[68] Lucké B (1944). The Pathology of Fatal Epidemic Hepatitis. Am J Pathol.

[69] Tandon BN, Bernauau J, O’Grady J (1999). Recommendations of the International Association for the Study of the Liver Subcommittee on nomenclature of acute and subacute liver failure. J Gastroenterol Hepatol.

[70] Polson J, Lee WM, Liver D (2005). AASLD position paper: the management of acute liver failure. Hepatology.

