# Supplementary material for: Massive hepatic necrosis-associated acute liver failure
Source: eGastroenterology. 2026 Jan 9;4(1):e100217. doi: 10.1136/egastro-2025-100217 (PMC12815235; doi:10.1136/egastro-2025-100217)
Supplement: Supplementary file 1 [file egastro-2025-100217.reviewer_comments.pdf]

PEER REVIEW HISTORY

eGastroenterology publishes all reviews undertaken for accepted manuscripts. Reviewers are asked to complete a checklist review form and are provided with free text boxes to elaborate on their assessment. These free text comments are reproduced below.

ARTICLE DETAILS

**TITLE (PROVISIONAL)**	Pathophysiology of massive hepatic necrosis-dependent acute liver failure
**AUTHORS**	Lin, Tao; Tong, Chenhao; Liebe, Roman; Weng, Honglei

VERSION 1 - REVIEW

**REVIEWER NAME**	***Ni, Hong-Min***
**REVIEWER AFFILIATION**	The University of Kansas Medical Center, Department of Pharmacology, Toxicology and Therapeutics
**REVIEWER CONFLICT OF INTEREST**	No
**AI DISCLOSURE**	No, I have not used AI
**DATE REVIEW RETURNED**	20-Apr-2025

**GENERAL COMMENTS**	The authors discussed the current understanding and advancements related to massive hepatic necrosis (MHN) associated with acute liver failure (ALF), focusing on the role and mechanisms of liver progenitor cells (LPCs). This topic is both intriguing and significant. Overall, the manuscript is well written, but a few additional clarifications could enhance its scope and quality.<br>Specific comments:<br>1. The authors state that “MHN” is not the same as “necrosis” (Page 10, line 31). This point requires further clarification to prevent confusion. It would be beneficial to briefly discuss different types of cell death and clearly differentiate “MHN” from other forms of cell death. Relevant details can be found in a recent review article (PMID: 40199509). The authors might consider commenting on the similarities and differences between apoptosis-induced ALF and MHN-ALF.<br>2. On Page 11, line 29, the statement “the definition of LPC remains controversial” appears redundant when compared to Page 12, line 21, which states, “the existence and definition of LPCs has been controversial in the scientific community.”<br>3. In the section titled “How can the smallest cholangiocytes proliferate in such harsh conditions and mediate liver regeneration?”, the authors mention that TGF-beta, EGF, and HGF promote LPC proliferation under ALF conditions. They also reference their own unpublished data to support this viewpoint. However, in cases of severe alcohol-associated hepatitis (which may differ from MHN-ALF), LPC or ductular reactions have been well documented (PMID: 36999531, 31311938). Both TGF-beta and EGF promote hepatocyte dedifferentiation and the loss of hepatocyte identity (PMID: 31311938).<br>4. The authors may want to add a discussion to clarify these varying contexts. The authors note that MHN-ALF differs from acetaminophen-induced liver failure. More details should be

	provided to explain the differences between MHN-ALF and acetaminophen-induced necrosis and liver failure. In addition to xenobiotic-induced liver failure, the authors could also discuss ConA-induced necrosis and liver injury. In both acetaminophen and ConA-induced acute liver failure, a new type of migratory hepatocyte, either ANXA2 (PMID: 38693268) positive or SOX9 positive, is critical for liver repair (PMCID: PMC10378165). This should be included in the discussion.<br>5. Minor point: Some of the abbreviations were not defined.

**REVIEWER NAME**	***Yin, Xiao-Ming***
**REVIEWER AFFILIATION**	Tulane University of Louisiana, Pathology
**REVIEWER CONFLICT OF INTEREST**	No
**AI DISCLOSURE**	No, I have not used AI
**DATE REVIEW RETURNED**	22-Apr-2025

**GENERAL COMMENTS**	This manuscript is comprehensive and insightful, addressing the distinct pathophysiology of acute liver failure (ALF) driven by massive hepatic necrosis (MHN). The authors combined pathological data with recent advances in liver progenitor cell (LPC) biology and regenerative mechanisms, which are informative for both clinicians and researchers. Recent zebrafish studies are cited to discuss LPC-to-hepatocyte differentiation and liver regeneration under severe liver injury. However, the manuscript could be improved further with clarification of the following points and with more discussion of translational implications for therapy and diagnosis.<br>Major Comments:<br>1. This manuscript involves ALF, ACLF, MHN and SMHN. It is easy to understand the difference between LAF and ACLF. Based on the main content of this manuscript, the authors should further clarify the difference between MHN and SMHN, with more discussion.<br>2. In "How does MHN occur" section, the manuscript focuses heavily on humoral and B-cell immunity (specific defenses) in MHN. It lacks discussion on the role of innate immune responses (Kupffer cells, NK cells or cytokine storms) which may also play key roles in hepatocyte death and LPC activation.<br>3. In "How can the severely damaged liver maintain liver function after MHN" section, the historical definition of LPCs is well covered, and the relationship between LPCs and cholangiocytes is well discussed. However, the dynamic plasticity between hepatocytes and cholangiocytes during regeneration in humans versus zebrafish needs clearer discussion. For example, is transdifferentiation equally possible in humans?<br>4. Although zebrafish is a widely used model, the evolutionary differences (e.g., absence of Kupffer cells) can be key limitations in the use of this model. Please include more findings from other models, such as the rodent model, which can further enhance the confidence in the conclusion drawn by this manuscript.<br>5. The discussion on FOXA2 is interesting, but it lacks the references to support the discussion. Is it discussed with unpublished data? The discussion should either be referenced or clearly indicated as a hypothesis.<br>6. While the manuscript is rich in content, more emphasis on how to guide therapeutic strategies (such as targeting TGF-β, BET inhibitors and HDAC1 inhibitors) in ALF patients would be more clinically interesting.

	Minor Comments:<br>1. Figure 1 is informative but confusing. Please simplify it or add additional panels to clarify the complex relationships between panel B and C.<br>2. There are some minor typographical errors, such as duplicated abbreviations of "STAT3" on page 3. Please check the whole manuscript to correct any typing errors and typographical errors.

**REVIEWER NAME**	***Feng, Dechun***
**REVIEWER AFFILIATION**	National Institutes of Health, National Institue on Alcohol Abuse and Alcoholism
**REVIEWER CONFLICT OF INTEREST**	No
**AI DISCLOSURE**	No, I have not used AI
**DATE REVIEW RETURNED**	29-Apr-2025

**GENERAL COMMENTS**	In this review, the authors summarize recent progress on how liver progenitor cells (LPCs) contribute to massive hepatic necrosis (MHN). They provide insightful information about the pathophysiology of MHN-dependent acute liver failure (ALF). The review is concise and well-organized. Here are some comments:<br>1. It would be better to separate Figure 1 into two figures: one focusing on ALF and the other on LPCs. Both figures could include more detailed information.<br>2. In this review, the authors discuss the unique features of LPCs, including how they survive and contribute to liver regeneration in the harsh environment of MHN patients. A related question is whether mature hepatocytes de-differentiate into LPCs or LPC-like cells during MHN. If so, what role do these cells play in liver regeneration?<br>3. In addition to possible LPC-to-hepatocyte differentiation, can LPCs secrete growth factors or other molecules that interact with other cell types such as hepatocytes, macrophages, and hepatic stellate cells?<br>4. With recent advances in single-cell sequencing, are there any studies that provide reliable datasets on LPCs in MHN?

VERSION 1 – AUTHOR RESPONSE

Reviewer: 1

The authors discussed the current understanding and advancements related to massive hepatic necrosis (MHN) associated with acute liver failure (ALF), focusing on the role and mechanisms of liver progenitor cells (LPCs). This topic is both intriguing and significant. Overall, the manuscript is well written, but a few additional clarifications could enhance its scope and quality.

Answer: We thank the reviewer for these positive comments. We are happy to clarify her/his concerns.

Specific comments:

1. The authors state that “MHN” is not the same as “necrosis” (Page 10, line 31). This point requires further clarification to prevent confusion. It would be beneficial to briefly discuss different types of cell death and clearly differentiate “MHN” from other forms of cell death. Relevant details can be found in a recent review article (PMID: 40199509). The authors might consider commenting on the similarities and differences between apoptosis-induced ALF and MHN-ALF.

Answer: We thank the reviewer for these constructive comments! As suggested, we modified descriptions of hepatocyte death in MHN-associated ALF in the revised manuscript (page 9-10):

“Hepatocyte death can occur by different mechanisms depending on disease conditions, including necrosis, apoptosis, necroptosis, autophagy, pyroptosis, and ferroptosis[26]. According to modern definitions of cell death, the term “massive hepatic necrosis” is misleading, as the nature of hepatocyte death in MHN is not exclusively necrosis. The term “massive necrosis” was first used in 1939 to describe severe liver injury in rats fed with Vitamin B6-deficient diet[27]. At that time, the concept of apoptosis and other cell death mechanisms had not yet been proposed. The Lucke and Mallory paper described virtually every pathologic feature of MHN[10]. Based on their observations, Lefkowitch concluded that “the severe form of confluent necrosis in which the large majority of hepatocytes have undergone cell death by either necrosis, apoptosis, or a combination of both (necroptosis)”[10]. Our previous study demonstrated hepatocyte apoptosis characterized by cleaved-caspase 3-positive cells in MHN[28]. However, few studies have confirmed necroptosis in patients with MHN-associated ALF to date.”

2. On Page 11, line 29, the statement “the definition of LPC remains controversial” appears redundant when compared to Page 12, line 21, which states, “the existence and definition of LPCs has been controversial in the scientific community.”

Answer: Thanks! As suggested, we have deleted the statement “the definition of LPC remains controversial” in the revised version of the manuscript.

3. In the section titled “How can the smallest cholangiocytes proliferate in such harsh conditions and mediate liver regeneration?”, the authors mention that TGF-beta, EGF, and HGF promote LPC proliferation under ALF conditions. They also reference their own unpublished data to support this viewpoint. However, in cases of severe alcohol-associated hepatitis (which may differ from MHN-ALF), LPC or ductular reactions have been well documented (PMID: 36999531, 31311938). Both TGF-beta and EGF promote hepatocyte dedifferentiation and the loss of hepatocyte identity (PMID: 31311938).

Answer: Thanks for the question. As the reviewer noted, severe alcohol-associated hepatitis differs from MHN-associated ALF. Classic ALF is clinical syndrome resulting from MHN (Lucke and Mallory. Am J Pathol 1946; O’Grady JG, et al. Lancet 1993). Based on the presence of MHN, ALF is induced by multiple etiologies, which do not comprise alcohol (see a table below, copied from Maiwall R, et al. Lancet 2024). In severe alcohol-associated hepatitis, MHN does not occur, and most hepatocytes remain viable. Instead, alcohol toxicity and fibrosis induce parenchymal hypoxia, leading to ductular metaplasia, which is defined as Ductular reaction (DR) type 2B by Desmet (Desmet VJ. Virchows Arch 2011). Meanwhile, Desmet defined smallest cholangiocytes (LPCs)-dependent DR as type 3 (Desmet VJ. Virchows Arch 2011). As the reviewer mentioned, multiple studies demonstrate the role of TGF-β and EGF in hepatocyte de-differentiation. Given that the current review focuses on MHN-associated ALF, we do not discuss different DR types.

***Panel:* Cause of acute liver failure**

Viral cause

- Hepatotropic viruses
  - Hepatitis A virus
  - Hepatitis E virus
  - Hepatitis B virus with or without hepatitis D virus
  - Hepatitis D virus
- Non-hepatotropic viruses
  - Herpes simplex virus
  - Varicella zoster virus
  - Epstein–Barr virus
  - Dengue virus
  - Adenovirus
  - COVID-19 virus
  - Cytomegalovirus

Drugs and toxins

- Paracetamol
- Amanita phalloides
- Anti-tuberculous drugs (especially isoniazid)
- Complementary and alternative medicines including herbal-induced liver injury
- Nitrofurantoin
- Phenytoin
- Propylthiouracil
- Yellow phosphorus
- Immune checkpoint inhibitors

Metabolic cause

- Wilson's disease

Other causes

- Autoimmune hepatitis
- Acute fatty liver of pregnancy
- Haemolysis elevation in liver enzymes and low platelet syndrome
- Ischaemic hepatitis
- Tumour infiltrates in the liver
- Hepatic venous outflow tract obstruction
- Haemophagocytic lymphohistiocytosis
- Budd–Chiari syndrome

Maiwall R, et al. Lancet 2024.

4. The authors may want to add a discussion to clarify these varying contexts. The authors note that MHN-ALF differs from acetaminophen-induced liver failure. More details should be provided to explain the differences between MHN-ALF and acetaminophen-induced necrosis and liver failure. In addition to xenobiotic-induced liver failure, the authors could also discuss ConA-induced necrosis and liver injury. In both acetaminophen and ConA-induced acute liver failure, a new type of migratory hepatocyte, either ANXA2 (PMID: 38693268) positive or SOX9 positive, is critical for liver repair (PMCID: PMC10378165). This should be included in the discussion.

Answer: We understand the reviewer's concerns. Massive hepatic necrosis (MHN) is defined as parenchymal loss of >90% of the entire liver by Hanau et al (Hanau C, et al. Hepatology 1995), or hepatocyte loss of 60-70% by other pathologists (Alastair,D.,et al. *MacSween's Pathology of the Liver, 6th Edn. 2012*). The extent of hepatocyte loss is critical for extensive LPC activation. Katoonizadeh et al. reported that a 50% loss of hepatocytes constitutes the threshold for extensive

LPC activation (Katoonizadeh A, et al. Liver Int 2006). According to the classic MHN definition, MHN occurs only in cases of HAV, HBV, HEV, and certain specific drugs. However, ALF itself can be induced by various etiologies, including other viral infections, APAP, and ConA in humans and animals, (Lefkowitch JH. Adv Anat Pathol 2016). Parenchymal loss induced by APAP and ConA damages hepatocytes at zone 3 of the lobes but usually does not result in a 50% loss of hepatocytes. In these cases, liver regeneration in APAP- and ConA-associated ALF depends on the remaining hepatocytes rather than on LPCs. This is why APAP and ConA rodent models do not mimic MHN-associated ALF (Lin T, et al. Biomolecules 2022). In an elegant review, Lefkowitch described hepatocyte death patterns across different etiologies of ALF (see the cartoon below).

We appreciate the important work published in Nature and J Clin Invest (PMID: 38693268 and PMCID: PMC10378165), which investigated mechanisms underlying APAP- and ConA-associated ALF. As this review focuses exclusively on MHN-associated ALF, we have not discussed these publications.

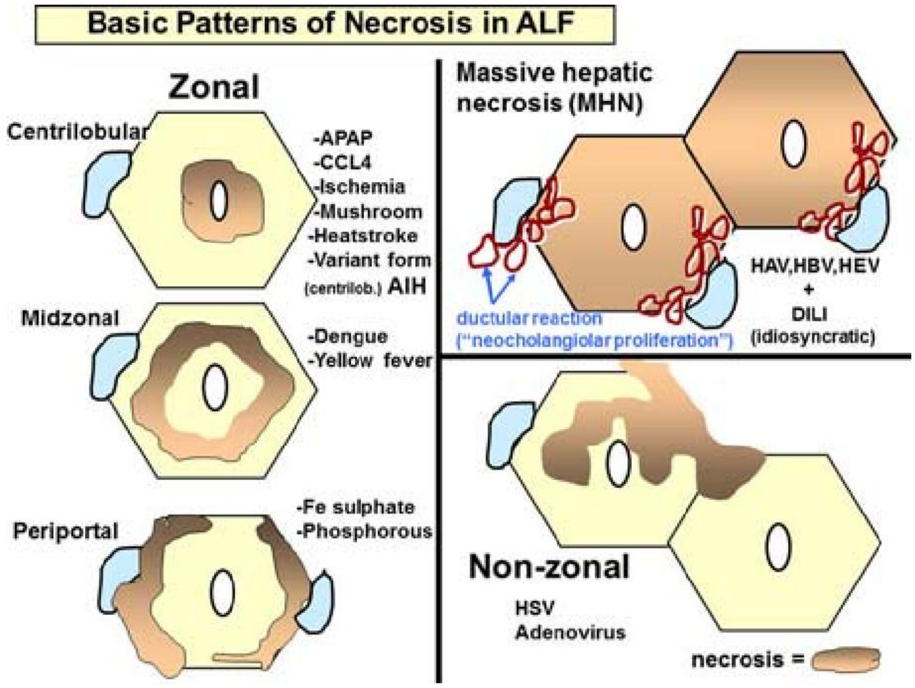

Lefkowitch JH. Adv Anat Pathol 2016

5. Minor point: Some of the abbreviations were not defined.

Answer: We have defined all abbreviations in the revised version of the manuscript.

Reviewer: 2

Comments to the Author

This manuscript is comprehensive and insightful, addressing the distinct pathophysiology of acute liver failure (ALF) driven by massive hepatic necrosis (MHN). The authors combined pathological data

with recent advances in liver progenitor cell (LPC) biology and regenerative mechanisms, which are informative for both clinicians and researchers. Recent zebrafish studies are cited to discuss LPC-to-hepatocyte differentiation and liver regeneration under severe liver injury. However, the manuscript could be improved further with clarification of the following points and with more discussion of translational implications for therapy and diagnosis.

Answer: We thank the reviewer for these positive comments. We are happy to clarify her/his concerns.

Major Comments:

1. This manuscript involves ALF, ACLF, MHN and SMHN. It is easy to understand the difference between LAF and ACLF. Based on the main content of this manuscript, the authors should further clarify the difference between MHN and SMHN, with more discussion.

Answer: In the revised version of the manuscript, we explain the differences between MHN and SMHN (page 5):

"Both MHN and SMHN are morphological features of ALF (formerly termed fulminant liver failure[11]. In liver pathology textbooks, MHN has been defined as extensive, diffuse panlobular (panacinar) and multilobular necrosis >60–70% of the entire liver upon examination via explant, autopsy, or clinical visualization. In contrast, SMHN describes lesions with global necrosis of 30–70% of the entire liver[11]. According to this definition, the difference between MHN and SMHN is reflected solely in the necrotic areas. Notably, MHN can evolute into SMHN over time. In ALF cases with clinical course exceeding 1-2 months, areas of necrosis become significantly reduced due to regeneration. Lefkowitch classified these cases as SMHN[10]."

2. In "How does MHN occur" section, the manuscript focuses heavily on humoral and B-cell immunity (specific defenses) in MHN. It lacks discussion on the role of innate immune responses (Kupffer cells, NK cells or cytokine storms) which may also play key roles in hepatocyte death and LPC activation.

Answer: We understand the reviewer's concern. Innate immune response, particularly mediated by macrophages, indeed plays a critical role in ALF patients following MHN. However, no evidence demonstrates that innate immunity contributes to the occurrence of MHN. Therefore, we do not discuss the role of innate immune response in the "How does MHN occur?" section. The role of macrophage-derived cytokines (e.g., TGF-β and HGF) has been discussed in the "How can the smallest cholangiocytes proliferate in such harsh conditions and mediate liver regeneration?" section.

3. In "How can the severely damaged liver maintain liver function after MHN" section, the historical definition of LPCs is well covered, and the relationship between LPCs and cholangiocytes is well discussed. However, the dynamic plasticity between hepatocytes and cholangiocytes during regeneration in humans versus zebrafish needs clearer discussion. For example, is transdifferentiation equally possible in humans?

Answer: This is a critical point in LPC biology. Reviewer-3 also asked a similar question. We explain this issue in the revised version of the manuscript (page 11-13).

"Liver progenitor cells belong to a tissue stem cell category. In humans, there is a fundamental covenant among cells: (1) Somatic cells stop reproduction and perform unique functions; (2) Germ cells propagate the genes; and (3) Stem cells repair tissues[34]. Some organs (e.g., skin and intestine)

possess unique organ-specific stem cells, whereas the liver does not[34]. However, this does not mean that the liver lacks stem-like cells to perform repairs in cases of severe liver diseases. Since Shinya Yamanaka and colleagues discovered that mature cells can be reprogrammed to become pluripotent, a clear boundary between somatic cells and stem cells has ceased to exist[35]. When provided with the necessary transcription factors under special conditions (e.g., forced transcription factor expression in cultured somatic cells), different cells can reprogram into each other through differentiation, transdifferentiation, and rejuvenation[36]. However, somatic cells cannot reprogram spontaneously in vivo due to physiological and pathophysiological limitations.

In adult livers, both hepatocytes and cholangiocytes can transdifferentiate into each other depending on disease conditions and repair demands[37]. In most studies, liver cells simultaneously expressing both hepatocyte markers (e.g., HNF4α, HNF1α, CK8, and CK18) and cholangiocyte markers (SOX9, CK7, and CK19) are termed as liver progenitor cells. Such cells are rarely observed in a normal liver. However, in various liver diseases, hepatocytes may express biliary transcription factors (e.g., SOX9) and biliary keratin (CK7 and CK19), while the smallest cholangiocytes residing in ductules and canals of Hering are can express hepatocyte transcription factors (e.g., HNF4α and HNF1α) and hepatic keratin (CK8 and CK18). The former is often observed in cholestasis. A classic example is primary biliary cholangitis (PBC). In longstanding PBC-dependent cholestasis, the neoductules, lined by cuboidal or flattened cells, are thought to arise from de-differentiation or metaplasia of periportal hepatocytes, and to a lesser extent, from proliferation of pre-existing ductules, depending on the degree of biliary obstruction (see a PBC patient presented in Figure 10.20 of the textbook[11]). Here, periportal hepatocytes act as LPCs by co-expressing biliary markers (CK7 and CK19) to repair damaged bile ducts. Similar phenomena have also been demonstrated in experimental animals fed with 3,5-diethoxycarbonyl-1,4-dihydrocollidine (DDC) or choline-deficient ethionine-supplemented (CDE) diet[38-40].

In contrast to hepatocyte de-differentiation in cholestasis, the smallest cholangiocytes expressing hepatocyte phenotypes are commonly observed in patients with MHN-dependent ALF[33,37]. In extremely severe MHN cases, no hepatocytes remain in ALF patients (see the patient-3[12] and the Patient-1[41]). In such a case, de-differentiation of mature hepatocytes is not possible since hepatocytes no longer exist. What about MHN-associated ALF patients retaining 30% hepatocytes? Do these remaining hepatocytes de-differentiate into LPCs or LPC-like cells? We propose the following explanations: (1) In the liver, hepatocytes are the primary cells performing functions essential for systemic homeostasis, while other liver cells act as supportive cells that enable hepatocyte function[42]. Following MHN, the remaining fewer than 30% of hepatocytes are insufficient to produce sufficient quantities of essential liver proteins. In this extreme scenario, the optimal strategy for patient survival is to let remaining hepatocytes produce liver function proteins maximally, while activating the second pathway of liver regeneration (LPC activation) to compensate for hepatocyte functions. (2) Theoretically, remaining hepatocytes could increase their numbers through proliferation. However, hepatocyte proliferation in MHN-associated ALF does not occur. In a recent study, we found that high levels of HGF-p-MET-p-STAT3 and EGF-p-EGFR-p-ERK signaling are active only in activated LPCs, not in remaining hepatocytes. These results indicate that remaining hepatocytes lack sensitivity to growth factor stimulation in this context. (3) Cell differentiation and reprogramming cannot be finalized in one cell cycle[43]. Ng and Gurdon reported that cell memory persist through 24 cell cycles without transcription, indicating that lineage change requires multiple cell cycles[44]. In MHN-dependent ALF, LPC-derived intermediate hepatocytes appear approximately three weeks after disease onset[14,32]. Therefore, proliferation is a prerequisite for cell differentiation. As mentioned, remaining hepatocytes do not proliferate in MHN-dependent ALF. This explains their inability to initiate de-differentiation in this harsh disease environment.

Collectively, LPC activation, hepatocyte function performance, and differentiation into mature hepatocytes are critical to rescue patients' lives and recover liver architecture/function in MHN-dependent ALF. Mature hepatocyte de-differentiation is therefore not viable. Therefore, the 2004

nomenclature clearly highlights that LPCs are smallest cholangiocytes residing in the Canal of Hering and the smallest biliary tree branches[33,45]."

4. Although zebrafish is a widely used model, the evolutionary differences (e.g., absence of Kupffer cells) can be key limitations in the use of this model. Please include more findings from other models, such as the rodent model, which can further enhance the confidence in the conclusion drawn by this manuscript.

Answer: It is true that zebrafish do not have Kupffer cells. Unfortunately, no rodent model currently exists that replicates MHN-associated ALF. Without intensive life support, rodents cannot survive the initial phase of MHN as patients can. We explained this limitation in a previous review (see Ref. 29).

5. The discussion on FOXA2 is interesting, but it lacks the references to support the discussion. Is it discussed with unpublished data? The discussion should either be referenced or clearly indicated as a hypothesis.

Answer: When we prepare the revised version of the manuscript, the two unpublished data have been published in bioRxiv. In the revised version of the manuscript, they are referenced as Ref. 43 and 56 respectively.

6. While the manuscript is rich in content, more emphasis on how to guide therapeutic strategies (such as targeting TGF-β, BET inhibitors and HDAC1 inhibitors) in ALF patients would be more clinically interesting.

Answer: Thanks for the suggestion. We are also interested in novel therapeutic strategies for MHN-associated ALF. Compared to other types of ALF (e.g., APAP- induced), most important studies on MHN-associated ALF were conducted decades ago. Given the lack of rodent models, detailed molecular mechanisms underlying this syndrome have primarily been investigated in clinical observation and in zebrafish, including the effect of TGF-β, BET, and HDAC1 on ALF progression. However, all these factors exert broad systemic effects rather than being liver-specific, limiting their clinical translatability. Therefore, the review focuses exclusively on pathophysiology and temporarily excludes clinical treatment considerations.

Minor Comments:

1. Figure 1 is informative but confusing. Please simplify it or add additional panels to clarify the complex relationships between panel B and C.

Answer: As suggested by reviewer-2 and reviewer-3, we update a new figure in the revised version of the manuscript.

2. There are some minor typographical errors, such as duplicated abbreviations of "STAT3" on page 3. Please check the whole manuscript to correct any typing errors and typographical errors.

Answer: Thanks! We have checked and corrected the typing errors and typographical errors in the revised version of the manuscript.

Reviewer: 3

Comments to the Author

In this review, the authors summarize recent progress on how liver progenitor cells (LPCs) contribute to massive hepatic necrosis (MHN). They provide insightful information about the pathophysiology of MHN-dependent acute liver failure (ALF). The review is concise and well-organized. Here are some comments:

1. It would be better to separate Figure 1 into two figures: one focusing on ALF and the other on LPCs. Both figures could include more detailed information.

Answer: We thank the reviewer for the suggestion. The similar suggestion was also proposed by the reviewer-2. In the revised version of the manuscript, we provide a new Figure 1for evaluation.

2. In this review, the authors discuss the unique features of LPCs, including how they survive and contribute to liver regeneration in the harsh environment of MHN patients. A related question is whether mature hepatocytes de-differentiate into LPCs or LPC-like cells during MHN. If so, what role do these cells play in liver regeneration?

Answer: We appreciate this constructive and key question. Clarifying "Whether mature hepatocytes de-differentiate into LPCs or LPC-like cells during MHN?" is very helpful to understand what LPCs are. Therefore, we discuss this pivotal question in the revised version of the manuscript on page 11-13:

"Liver progenitor cells belong to a tissue stem cell category. In humans, there is a fundamental covenant among cells: (1) Somatic cells stop reproduction and perform unique functions; (2) Germ cells propagate the genes; and (3) Stem cells repair tissues[34]. Some organs (e.g., skin and intestine) possess unique organ-specific stem cells, whereas the liver does not[34]. However, this does not mean that the liver lacks stem-like cells to perform repairs in cases of severe liver diseases. Since Shinya Yamanaka and colleagues discovered that mature cells can be reprogrammed to become pluripotent, a clear boundary between somatic cells and stem cells has ceased to exist[35]. When provided with the necessary transcription factors under special conditions (e.g., forced transcription factor expression in cultured somatic cells), different cells can reprogram into each other through differentiation, transdifferentiation, and rejuvenation[36]. However, somatic cells cannot reprogram spontaneously in vivo due to physiological and pathophysiological limitations.

In adult livers, both hepatocytes and cholangiocytes can transdifferentiate into each other depending on disease conditions and repair demands[37]. In most studies, liver cells simultaneously expressing both hepatocyte markers (e.g., HNF4α, HNF1α, CK8, and CK18) and cholangiocyte markers (SOX9, CK7, and CK19) are termed as liver progenitor cells. Such cells are rarely observed in a normal liver. However, in various liver diseases, hepatocytes may express biliary transcription factors (e.g., SOX9) and biliary keratin (CK7 and CK19), while the smallest cholangiocytes residing in ductules and canals of Hering are can express hepatocyte transcription factors (e.g., HNF4α and HNF1α) and hepatic keratin (CK8 and CK18). The former is often observed in cholestasis. A classic example is primary biliary cholangitis (PBC). In longstanding PBC-dependent cholestasis, the neoductules, lined by cuboidal or flattened cells, are thought to arise from de-differentiation or metaplasia of periportal

hepatocytes, and to a lesser extent, from proliferation of pre-existing ductules, depending on the degree of biliary obstruction (see a PBC patient presented in Figure 10.20 of the textbook[11]). Here, periportal hepatocytes act as LPCs by co-expressing biliary markers (CK7 and CK19) to repair damaged bile ducts. Similar phenomena have also been demonstrated in experimental animals fed with 3,5-diethoxycarbonyl-1,4-dihydrocollidine (DDC) or choline-deficient ethionine-supplemented (CDE) diet[38-40].

In contrast to hepatocyte de-differentiation in cholestasis, the smallest cholangiocytes expressing hepatocyte phenotypes are commonly observed in patients with MHN-dependent ALF[33,37]. In extremely severe MHN cases, no hepatocytes remain in ALF patients (see the patient-3[12] and the Patient-1[41]). In such a case, de-differentiation of mature hepatocytes is not possible since hepatocytes no longer exist. What about MHN-associated ALF patients retaining 30% hepatocytes? Do these remaining hepatocytes de-differentiate into LPCs or LPC-like cells? We propose the following explanations: (1) In the liver, hepatocytes are the primary cells performing functions essential for systemic homeostasis, while other liver cells act as supportive cells that enable hepatocyte function[42]. Following MHN, the remaining fewer than 30% of hepatocytes are insufficient to produce sufficient quantities of essential liver proteins. In this extreme scenario, the optimal strategy for patient survival is to let remaining hepatocytes produce liver function proteins maximally, while activating the second pathway of liver regeneration (LPC activation) to compensate for hepatocyte functions. (2) Theoretically, remaining hepatocytes could increase their numbers through proliferation. However, hepatocyte proliferation in MHN-associated ALF does not occur. In a recent study, we found that high levels of HGF-p-MET-p-STAT3 and EGF-p-EGFR-p-ERK signaling are active only in activated LPCs, not in remaining hepatocytes. These results indicate that remaining hepatocytes lack sensitivity to growth factor stimulation in this context. (3) Cell differentiation and reprogramming cannot be finalized in one cell cycle[43]. Ng and Gurdon reported that cell memory persist through 24 cell cycles without transcription, indicating that lineage change requires multiple cell cycles[44]. In MHN-dependent ALF, LPC-derived intermediate hepatocytes appear approximately three weeks after disease onset[14,32]. Therefore, proliferation is a prerequisite for cell differentiation. As mentioned, remaining hepatocytes do not proliferate in MHN-dependent ALF. This explains their inability to initiate de-differentiation in this harsh disease environment.

Collectively, LPC activation, hepatocyte function performance, and differentiation into mature hepatocytes are critical to rescue patients' lives and recover liver architecture/function in MHN-dependent ALF. Mature hepatocyte de-differentiation is therefore not viable. Therefore, the 2004 nomenclature clearly highlights that LPCs are smallest cholangiocytes residing in the Canal of Hering and the smallest biliary tree branches[33,45]."

"Liver progenitor cells belong to a tissue stem cell category. In humans, there is a fundamental covenant among cells: (1) Somatic cells stop reproduction and perform unique functions; (2) Germ cells propagate the genes; and (3) Stem cells repair tissues[34]. Some organs (e.g., skin and intestine) possess unique organ-specific stem cells, whereas the liver does not[34]. However, this does not mean that the liver lacks stem-like cells to perform repairs in cases of severe liver diseases. Since Shinya Yamanaka and colleagues discovered that mature cells can be reprogrammed to become pluripotent, a clear boundary between somatic cells and stem cells has ceased to exist[35]. When provided with the necessary transcription factors under special conditions (e.g., forced transcription factor expression in cultured somatic cells), different cells can reprogram into each other through differentiation, transdifferentiation, and rejuvenation[36]. However, somatic cells cannot reprogram spontaneously in vivo due to physiological and pathophysiological limitations.

In adult livers, both hepatocytes and cholangiocytes can transdifferentiate into each other depending on disease conditions and repair demands[37]. In most studies, liver cells simultaneously expressing both hepatocyte markers (e.g., HNF4α, HNF1α, CK8, and CK18) and cholangiocyte markers (SOX9, CK7, and CK19) are termed as liver progenitor cells. Such cells are rarely observed in a normal liver.

However, in various liver diseases, hepatocytes may express biliary transcription factors (e.g., SOX9) and biliary keratin (CK7 and CK19), while the smallest cholangiocytes residing in ductules and canals of Hering are can express hepatocyte transcription factors (e.g., HNF4α and HNF1α) and hepatic keratin (CK8 and CK18). The former is often observed in cholestasis. A classic example is primary biliary cholangitis (PBC). In longstanding PBC-dependent cholestasis, the neoductules, lined by cuboidal or flattened cells, are thought to arise from de-differentiation or metaplasia of periportal hepatocytes, and to a lesser extent, from proliferation of pre-existing ductules, depending on the degree of biliary obstruction (see a PBC patient presented in Figure 10.20 of the textbook[11]). Here, periportal hepatocytes act as LPCs by co-expressing biliary markers (CK7 and CK19) to repair damaged bile ducts. Similar phenomena have also been demonstrated in experimental animals fed with 3,5-diethoxycarbonyl-1,4-dihydrocollidine (DDC) or choline-deficient ethionine-supplemented (CDE) diet[38-40].

In contrast to hepatocyte de-differentiation in cholestasis, the smallest cholangiocytes expressing hepatocyte phenotypes are commonly observed in patients with MHN-dependent ALF[33,37]. In extremely severe MHN cases, no hepatocytes remain in ALF patients (see the patient-3[12] and the Patient-1[41]). In such a case, de-differentiation of mature hepatocytes is not possible since hepatocytes no longer exist. What about MHN-associated ALF patients retaining 30% hepatocytes? Do these remaining hepatocytes de-differentiate into LPCs or LPC-like cells? We propose the following explanations: (1) In the liver, hepatocytes are the primary cells performing functions essential for systemic homeostasis, while other liver cells act as supportive cells that enable hepatocyte function[42]. Following MHN, the remaining fewer than 30% of hepatocytes are insufficient to produce sufficient quantities of essential liver proteins. In this extreme scenario, the optimal strategy for patient survival is to let remaining hepatocytes produce liver function proteins maximally, while activating the second pathway of liver regeneration (LPC activation) to compensate for hepatocyte functions. (2) Theoretically, remaining hepatocytes could increase their numbers through proliferation. However, hepatocyte proliferation in MHN-associated ALF does not occur. In a recent study, we found that high levels of HGF-p-MET-p-STAT3 and EGF-p-EGFR-p-ERK signaling are active only in activated LPCs, not in remaining hepatocytes. These results indicate that remaining hepatocytes lack sensitivity to growth factor stimulation in this context. (3) Cell differentiation and reprogramming cannot be finalized in one cell cycle[43]. Ng and Gurdon reported that cell memory persist through 24 cell cycles without transcription, indicating that lineage change requires multiple cell cycles[44]. In MHN-dependent ALF, LPC-derived intermediate hepatocytes appear approximately three weeks after disease onset[14,32]. Therefore, proliferation is a prerequisite for cell differentiation. As mentioned, remaining hepatocytes do not proliferate in MHN-dependent ALF. This explains their inability to initiate de-differentiation in this harsh disease environment.

Collectively, LPC activation, hepatocyte function performance, and differentiation into mature hepatocytes are critical to rescue patients' lives and recover liver architecture/function in MHN-dependent ALF. Mature hepatocyte de-differentiation is therefore not viable. Therefore, the 2004 nomenclature clearly highlights that LPCs are smallest cholangiocytes residing in the Canal of Hering and the smallest biliary tree branches[33,45]."

3. In addition to possible LPC-to-hepatocyte differentiation, can LPCs secrete growth factors or other molecules that interact with other cell types such as hepatocytes, macrophages, and hepatic stellate cells?

Answer: Yes, LPC-producing growth factors, cytokines, or chemokines are critical in MHN-associated ALF pathophysiology. Our recent study provides preliminary results in this aspect. In this study, we performed a spatial transcriptome analysis for the tissue samples from 4 patients with MHN-associated ALF. We analyzed the cellular communications between LPCs, remaining hepatocytes, macrophages and activated HSCs in these patients. There were high levels of cell-cell interaction

counts and weights between LPCs, macrophages and HSCs. On the one hand, LPCs received multiple strong signals from surrounding cells, including TGF-β, activin, EGF, HGF, FGF, TWEAK and IGF. On the other hand, LPCs also sent strong signals to surrounding cells, including TGF-β, EGF, FGF, CXCL, CSF, and PDGF. Further cell-cell communication analysis revealed signal transduction direction between LPCs and surrounding cells. Macrophages and activated HSCs sent TGF-β, BMP, NODAL, EGF, HGF, FGF, TWEAK, IGF and CXCL to LPCs. LPCs sent TGF-β, activin, BMP, NODAL, EGF, HGF, FGF, CSF, PDGF and CXCL to macrophages and TGF-β, activin, BMP, NODAL, EGF, FGF, CSF, PDGF and CXCL to activated HSCs. In addition, LPCs produced TGF-β, activin, BMP, EGF, FGF, TWEAK, IGF and CXCL for autoregulation.

We have briefly discussed this issue on page 16 of the manuscript. More detailed information has been described in our preprint paper (doi: https://doi.org/10.1101/2025.06.04.657336).

4. With recent advances in single-cell sequencing, are there any studies that provide reliable datasets on LPCs in MHN?

Answer: Unfortunately, we currently cannot find any available liver single-cell sequencing database, which was performed in patients with MHN-associated ALF.

VERSION 2 - REVIEW

**REVIEWER NAME**	***Ni, Hong-Min***
**REVIEWER AFFILIATION**	The University of Kansas Medical Center, Department of Pharmacology, Toxicology and Therapeutics
**REVIEWER CONFLICT OF INTEREST**	No
**AI DISCLOSURE**	No, I have not used AI
**DATE REVIEW RETURNED**	16-Jun-2025

**GENERAL COMMENTS**	The revised manuscript addressed all the comments raised by this reviewer.

**REVIEWER NAME**	***Feng, Dechun***
**REVIEWER AFFILIATION**	National Institutes of Health, National Institue on Alcohol Abuse and Alcoholism
**REVIEWER CONFLICT OF INTEREST**	No
**AI DISCLOSURE**	No, I have not used AI
**DATE REVIEW RETURNED**	13-Jun-2025

**GENERAL COMMENTS**	I am satisfied with the revised version
